# Editorial: Bringing function to the forefront of cell therapy: how do we demonstrate potency?

**DOI:** 10.3389/fimmu.2024.1472727

**Published:** 2024-08-12

**Authors:** Alasdair R. Fraser, Stuart M. Curbishley, Andy Roemhild, Kwong Tsang

**Affiliations:** ^1^ Swarm Oncology Ltd., Glasgow, United Kingdom; ^2^ Adthera Bio Ltd., Birmingham, United Kingdom; ^3^ Berlin Center for Advanced Therapies, Charité Universitätsmedizin Berlin, Berlin, Germany; ^4^ Precision Biologics, Bethesda, MD, United States

**Keywords:** potency, cell therapy, assays, T cells, mesenchymal stem cell, NK cell

Cell-based treatments are a vital component of clinical immune therapies. These cell therapies encompass everything from haematopoietic stem cells, T cells, natural killer (NK) cells, dendritic cells and macrophages, to non-immune cells such as mesenchymal stromal cells (MSC) and endothelial cells. T cells have generated the greatest interest and development for therapeutic use through virus-specific Cytotoxic T Lymphocytes (CTL), Tumour-Infiltrating Lymphocytes (TIL), gamma-delta T cells, regulatory T cells and in particular CAR-T cells. MSC and endothelial cells also have enormous potential in tissue repair and regeneration.

The principal requirement linking these Advanced Therapeutic Medicinal Products (ATMPs) is that they are appropriately characterised. Regulators require evidence that target cells are suitable and safe for use, and that there are indicators of potency. The potency of the therapy depends upon the cell type involved but is defined as “the specific ability or capacity of the product, as indicated by appropriate laboratory tests or by adequately controlled clinical data obtained through the administration of the product in the manner intended, to effect a given result” (FDA guidelines). The key issue is which assays represent an appropriate test, so development of effective *in vitro* assays and surrogate markers of efficacy are essential to simplify the translation and use of new cellular therapies.

This Research Topic highlights new approaches for *in vitro* testing of cell therapy material and demonstrates the potential for surrogate markers to define Critical Quality Attributes of the therapy. The articles are written by leading practitioners in cell therapy and include an outstanding perspective piece by Lowdell and Weil which effectively summarises the topic and excellent reviews from three groups, assessing the use of potency assays and biomarkers (Capelli et al.), NK-based therapies in combination with antibodies (Fantini et al.) and cell therapies for treatment of COVID (Gonzaga et al.). In addition there are two key original research papers which describe the use of rapid assays for assessing MSC immunosuppression (Herzig et al.) and the development of comprehensive analytical approaches and surrogate assays for cytotoxicity assessment in virus-specific T cell therapy (Cooper et al.).

The Perspective from Lowdell and Weil perfectly captures the challenge of identifying appropriate methods to demonstrate function and potency. They discuss the issue of measuring function where the Mechanism of Action is not clear. This is particularly relevant when it comes to establishing defined values for Critical Quality criteria for ATMPs, noting that *in vitro* outcomes do not necessarily predict clinical efficacy. They conclude that it is essential to gather as much data as possible from pre-clinical steps for assays which provide clear and understandable information regarding the quality of the ATMP.

The review from Capelli et al. looks at ATMP potency in more detail and examines the various assays used to quantify function in a range of different modified (CAR-T, CAR-NK, TCR-T cells and iPSC) and unmodified cell therapies (lymphocytes, MSC, chondrocytes, epithelial cells and dendritic cells). They highlight the challenge of conventional cytotoxicity assays and suggest that surrogate markers of function (cytokine assays, tetramer labelling and ELISpot) may offer a suitable alternative if suitably validated.

The second review by Fantini et al. focuses on the potentiation of NK-based cell therapies for cancer treatment. They review the role of NK cells in adoptive cell therapy and the use of strategies (eg. cytokine stimulation, CAR-NK) to enhance anti-tumour effects. The main target of the review is the use of monoclonal antibodies in combination with NK cells to enhance functionality, either through checkpoint inhibition or by harnessing ADCC activity, approaches which show real promise in boosting anti-tumour responses.

Finally the review from groups led by Gonzaga et al. looks at the use of combined therapies to treat COVID, combining conventional treatments (cytokine inhibitors, dexamethasone and corticosteroids) with new cell therapies. Adoptive transfer of COVID-specific T cells to patients with severe COVID had no infusion-related issues and gave dose-dependent improvement in hospital discharge times. Treatment of severe COVID with MSC to restore lung function and minimise inflammatory damage demonstrated few adverse effects, and combination with other treatments may offer significant benefits. These reviews all add substantially to the understanding of cell therapies in clinical use and highlight the need for appropriate assays to determine the efficacy of cells for treatment of serious diseases.

The two original articles present compelling evidence for the use of surrogate assays to assess function and potency in two different cell types. The research from Andrew Cap’s lab (Herzig et al.) highlights the issue of quantifying MSC function. Assays of MSC potency focus on inhibition of T cell proliferation, both time-consuming and reflecting only one aspect of MSC activity. Their study investigates the use of rapid alternative assays. Inhibition of caspases did not produce consistent outcomes except after 24 hours, but suppression of phosphatidyl serine (PS) target cell externalisation and TNFa release were both robust indicators of immune suppression and therefore useful as surrogate assays for MSC function.

The article from Cooper et al. compares two manufacturing approaches for EBV-specific T cell therapies and uses comprehensive analysis to compare the outcomes in terms of final products. Multiparameter flow cytometry, t-SNE analysis and clonotyping defined the differentiation and specificity of these products and demonstrated that the new peptide-driven manufacturing method offers substantial benefits over conventional manufacture. A key aspect of the work was functional analysis which confirmed that TNFa/IFNg co-expression correlated closely with cytotoxicity and is a reliable surrogate assay.

These contributions confirm that potency is an ongoing challenge for cell therapy and that the development of effective assays of cellular function are central to the regulation and licencing of ATMPs. This Research Topic demonstrates that there are alternatives to conventional measures of potency which will drive faster, simpler and more quantitative assays for characterising new cell therapies.

## Author contributions

AF: Writing – original draft, Writing – review & editing. SC: Writing – review & editing. AR: Writing – review & editing. KT: Writing – review & editing.

